# Nanozyme-based biosensors for pathogenic microorganism detection: a mini-review

**DOI:** 10.3389/fmicb.2026.1994360

**Published:** 2026-09-30

**Authors:** Ting Yang, Yuting Ling, Enmei Zhang, Jin Li

**Affiliations:** College of Pharmacy and Bioengineering, Chongqing University of Technology, Chongqing, China

**Keywords:** catalytic mechanisms of nanozymes, clinical pathogens diagnosis, detection of foodborne bacteria, monitoring of environmental microorganisms, nanozyme biosensing platforms, nanozyme-based biosensors

## Abstract

Nanozyme technology has surfaced as a game-changing tool in the detection of pathogenic microorganisms, serving as an appealing substitute for traditional biosensing methods. This review first highlighted the unique catalytic mechanisms of nanozymes, including peroxidase-like, catalase-like, oxidase-like, and superoxide dismutase-like activities, which are fundamental to their usefulness. Second, we explored mainstream biosensing platforms, such as colorimetric/lateral flow assays, electrochemical sensors, fluorescent/dual-modal systems, and nanozyme-CRISPR synergistic approaches. Each of these systems benefited from the stability, tunability, and cost-effectiveness of nanozymes. Third, the applications of nanozyme-based biosensors in the diagnosis of clinical pathogens, the detection of foodborne bacteria, and the monitoring of environmental microorganisms were further analyzed. Finally, the future directions of nanozyme-based biosensors were discussed to advance their clinical and industrial translation.

## 1 Introduction

As pathogenic microorganisms, bacteria, viruses, fungi, and parasites pose a serious threat to public health and food safety. The European Rapid Alert System for Food and Feed has documented a substantial number of notifications concerning pathogenic microorganisms in food products, underscoring their prevalence and the associated risks (Eissa et al., 2025). In this context, early and accurate pathogen detection is indispensable for restraining infectious transmission, containing disease outbreaks, and establishing reliable prevention and control strategies. Traditional detection techniques, including microbial culture, polymerase chain reaction (PCR), and enzyme-linked immunosorbent assay, have served as fundamental tools in routine microbial diagnosis (Chang et al., 2025). Nevertheless, these conventional approaches suffer from inherent disadvantages including time-consuming procedures, heavy instrument reliance, and unsatisfactory on-site detection capability, which greatly restrict their practical applicability in resource-limited and field scenarios.

Owing to their unique catalytic activities and stability, nanozymes, synthetic nanomaterials with enzyme-like properties, have become a promising tool for pathogen detection compared to natural enzymes. Since the discovery of ferromagnetic nanoparticles exhibiting horseradish peroxidase-like activity in 2007, this rapidly growing field has made considerable progress, leading to a diverse array of applications across industrial, medical, and biological domains (Huang et al., 2019). The application of nanozymes in pathogenic detection is particularly significant given the growing need for rapid, sensitive, and economical diagnostic techniques. Moreover, nanozyme-based biosensors offer several critical advantages over traditional detection methods. They possess excellent potential to exceed the limitations of existing assays through innovative engineering techniques and integrated biosensing approaches.

Against this backdrop, this mini-review systematically summarizes the catalytic mechanisms of nanozymes, cutting-edge biosensing platforms, and their typical applications in pathogen detection, while also offering new insights based on the latest research in the field (Figure 1). Compared with previous related reviews, this mini-review emphasizes the integration of nanozymes with CRISPR-Cas and aptamer biosensing platforms for rapid detection of pathogenic microorganisms, particularly drug-resistant pathogens (Cheng et al., 2025; Ma et al., 2023). Moreover, this review covered publications mainly from 2023 to mid-2026, which offered timely and innovative insights into the rational design and practical application of pathogen detection using nanozyme-based biosensors.

**Figure 1:**
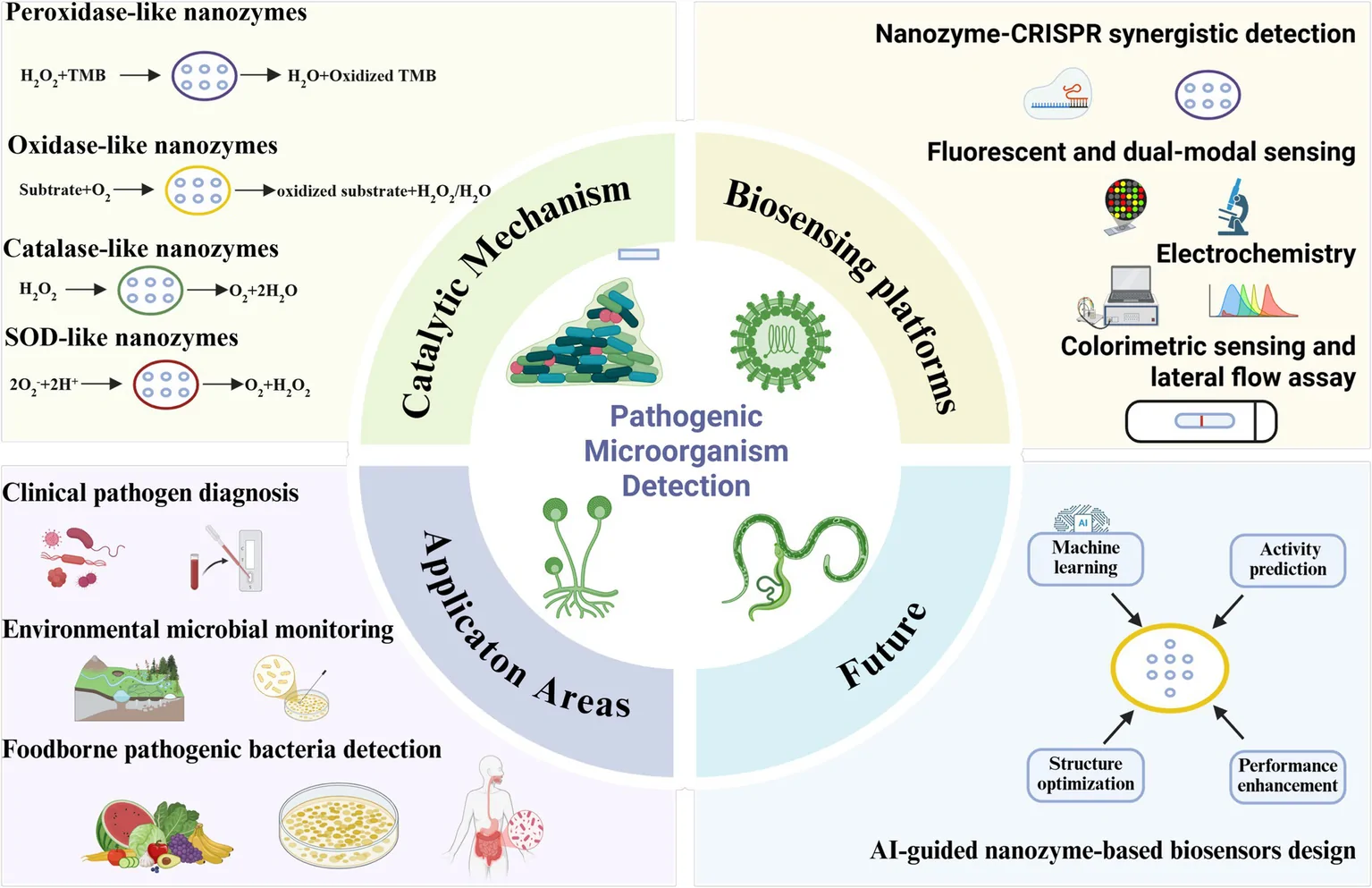
Schematic overview of nanozyme-based biosensors for pathogenic microorganism detection: catalytic mechanisms, biosensing platforms, application areas and future AI-assisted strategy. TMB, 3,3′,5,5′-tetramethylbenzidine; SOD, superoxide dismutase; LFA, lateral flow assay; CRISPR, clustered regularly interspaced short palindromic repeats; Created in https://BioRender.com.

## 2 The catalytic mechanisms of nanozymes

### 2.1 Peroxidase-like nanozymes

Nanozymes with peroxidase-like activity have become a significant asset in biosensing because they can catalyze substrate oxidation when hydrogen peroxide (H₂O₂) is present. This process converts pathogenic microorganism recognition events into measurable signals. Peroxidase-like nanozymes primarily function by producing reactive oxygen species (ROS), including hydroxyl radicals (•OH), which are crucial for the catalytic process. For instance, Fe-MOGs (metal–organic gels) have been shown to catalyze the breakdown of H₂O₂ into ROS through a Fenton-like reaction, significantly amplifying electrochemiluminescence signals for biosensing applications (Zong et al., 2021). The finding underscores the importance of ROS in the catalytic mechanisms of peroxidase-like nanozymes. The structural and electronic features of nanozymes are also vital for their catalytic performance. For example, single-atom Co-MoS₂ nanozymes demonstrate distinct catalytic pathways, with Co atoms favoring electron transfer pathways and MoS₂ depending on Fenton-like reactions (Wang et al., 2019). One core-shell type nanozyme has been reported to achieve enhanced peroxidase-like activity and improved stability through interfacial reactions between the core and shell materials (Wang et al., 2026; Bhagat et al., 2018). Furthermore, single-atom nanozymes were reported to feature metal active sites that are dispersed atomically (Kang et al., 2025). The nanozymes can precisely regulate the generation pathway, yield, and species of ROS through modulation of their local coordination environment. Moreover, through an artificial intelligence (AI)-powered graph neural network, an optimal Cu-BTC/Ti₃C₂Tₓ (2:1) nanozyme was screened out, which showed enhanced peroxidase-like activity with an ultralow *K*_m_ to H_2_O_2_ (Tong and Han, 2025).

### 2.2 Catalase-like nanozymes

The nanozymes with catalase-like activity, on the one hand, can transform H₂O₂ generated during pathogen metabolism into quantifiable O₂ signals. On the other hand, they can scavenge excess H₂O₂ background interference in the detection system. One of the key aspects of catalase-like nanozymes is their structural and compositional diversity, which significantly influences their catalytic activity. For instance, the study on Co₂V₂O₇ particles demonstrated that these nanozymes exhibit intrinsic multienzyme mimetic activities, including catalase-like activity, due to their unique granular structure synthesized via a hydrothermal method (Zhang et al., 2020). This structural advantage allows for efficient H₂O₂ decomposition, making them suitable for ultrasensitive biosensing applications. Owing to the presence of oxygen vacancies and electronic environment of the active sites, catalase-like nanozymes processed catalytic efficiency. For instance, Co₃O₄/CoFe₂O₄ hollow nanocubes with oxygen vacancies have demonstrated improved catalytic performance because of the synergistic interaction between the outer and inner oxygen and electron transfer between Co and Fe (Jiang et al., 2023). This highlights the importance of optimizing the electronic and surface characteristics of nanozymes to enhance catalytic activity.

### 2.3 Oxidase-like nanozymes

The nanozymes with oxidase-like activity catalyze substrates oxidation through molecular oxygen (O₂) acting as an electron acceptor, thereby eliminating the need for H₂O₂. One of the foundational studies in this area demonstrated that noble metal nanoparticles including gold (Au) and platinum (Pt), can act like the glucose oxidase enzyme by promoting glucose oxidation through a two-step reaction. This involves the dehydrogenation of glucose followed by the reduction of oxygen to H_2_O_2_ or water, depending on the metal used (Chen et al., 2021). This mechanism works similarly to that of natural glucose oxidase, highlighting the potential of noble metal nanozymes in biosensing applications. Further research has shown that the oxidase-like activity of nanozymes can be enhanced by modifying their surface structures or by using specific ligands. For instance, citrate-capped platinum nanoparticles demonstrated enhanced oxidase-like activity in certain chromogenic systems, which is attributed to simultaneous two-electron and four-electron reduction processes (Lin et al., 2021). This enhancement in activity is crucial for developing more sensitive and specific biosensors, as demonstrated by the detection of trypsin and its inhibitors using Cit-PtNPs. In conclusion, the oxidase-like nanozymes have complicated catalytic processes that influenced by various factors, including the type of metal, surface modifications, and structural properties.

### 2.4 Superoxide dismutase-like nanozymes

Owing to its capacity to convert superoxide radicals (O₂•^−^) into O₂ and H₂O₂, superoxide dismutase (SOD)-like nanozymes have become as a promising replacement for natural SODs. In pathogen detection, they can convert pathogen-triggered O₂•^−^ into H₂O₂, which then interacts with peroxidase-like nanozymes to produce detectable readouts. The catalytic mechanisms of SOD-like nanozymes are further elucidated through the study of various nanomaterials. For example, MoS2 nanozymes exhibit activities of multiple antioxidant enzymes, functioning as a self-cascade system to suppress the generation of intracellular ROS. The enhancement of the antioxidant defense system was achieved through the modulation of mitochondrial function and the removal of ROS (Zhang et al., 2021). To effectively scavenge O₂•^−^, similarly, a cerium-based metal–organic frameworks (Ce-MOFs) have been engineered to mimic SOD activity through a redox cycle between Ce(IV) and Ce(III) (Liu et al., 2022). These Ce-MOFs have demonstrated efficacy in ionizing radiation protection, highlighting their potential as radioprotectants. Moreover, platinum-based nanoenzymes, which exhibit dual activities of both SOD and CAT, were used to develop an SOD-CAT cascade system for eliminating various reactive oxygen species. For example, laminarin-modified Pt@LA nanozymes can sequentially degrade ROS to reduce oxidative stress (Guo et al., 2025).

## 3 Mainstream nanozyme biosensing platforms

### 3.1 Colorimetric sensing and lateral flow assay (LFA)

Colorimetric sensing and LFAs are the most widely used point-of-care testing (POCT) platforms, employing nanozymes’ catalytic activity to achieve visible changes in color without sophisticated instruments. However, the techniques still face inherent drawbacks. For example, due to the reliance on naked-eye signal interpretation, conventional detection methods have difficulty performing simultaneous analyses of various pathogenic microorganisms. Utilizing split aptamers and mannan oligosaccharide nanozymes, Li et al. developed a colorimetric biosensor for His-tagged food biomolecules, achieving a limit of detection (LOD) of 12.44 nM (Wei et al., 2025). In another report, Prussian blue (PB) nanozymes were implemented in a paper-based LFA for dopamine detection. This strategy yields an LOD of 0.59 μM and mitigates the intrinsic poor stability of PB in aqueous media (Zhang et al., 2023). Moreover, Wang et al. demonstrated that integration with smartphones further broadens the practical applicability of such assays. For example, the microcystin-leucine-arginine (MC-LR) can harm human health and the environment. A CeO₂-nanocage-based colorimetric LFA for MC-LR reaches an LOD of 0.66 pg./mL, where assay readouts are processed by a mobile app (Wang et al., 2024). Traditional detection methods such as culture and mass spectrometry, are time-intensive and hard to realize simultaneous rapid identification of multiple pathogens. Microfluidic arrays provide a viable option. Zhang et al. created a microfluidic array platform that with enhanced electrochemiluminescence by integrating of aptamer-functionalized DNA nanospheres (Zhang C. et al., 2025). This platform can simultaneously detect four pathogenic bacteria, including methicillin-resistant *Staphylococcus aureus* (MRSA), *Klebsiella pneumoniae*, *Pseudomonas aeruginosa*, and *S. aureus* with assay time only 45 min and a LOD ≤ 100 CFU/mL. Owing to the advantages of rapidity, instrument-free operation, and low cost, the colorimetric sensing and LFA platform is suitable for on-site microbial screening. However, major bottlenecks such as limited quantitative precision and interferences from matrix components need to be overcome.

### 3.2 Electrochemical nanozyme-based biosensing

Electrochemical biosensors use nanozymes to catalyze redox reactions, generating measurable current or potential signals. High sensitivity constitutes one of their major merits. For uric acid detection, a Co-based single-atom nanozyme delivers a broad linear range spanning 0.4—41,950 μM together with an LOD of 33.3 nM (Hu et al., 2020). Coupling nanozymes with nanochannels improves detection selectivity. For telomerase detection, a target-triggered growth strategy of Prussian blue nanoparticles (PBNPs) inside TiO₂ nanochannels delivers an LOD as low as 0.063 pM (Dai et al., 2020). Another study used Au-Pt bimetallic nanoparticles on carbon nanotubes (CNTs) as a NADH oxidase mimic, enabling xylose detection with an LOD of 1 μM (Song et al., 2020). Electrochemical biosensors also enable multi-analyte detection. For example, a dual-mode sensor for glucose using CuWO₄ NPs achieved an LOD of 1.5 μM (electrochemical) and 0.05 mM (colorimetric) (Zhang et al., 2022). Moreover, these platforms are also suitable for clinical applications. A biosensor for CEA using CuS@Pt-SA nanozymes achieved an LOD of 3 fM, enabling early cancer diagnosis (Xie et al., 2023). Another example, an amplification-free CRISPR-AsCas12a electrochemiluminescent biosensor was developed by Zhang et al., utilizing tetrahedral DNA nanostructures (Zhang X. et al., 2025). The effective detection of colorectal-cancer-associated *Fusobacterium nucleatum* was achieved with an LOD of 1 CFU/mL by using coral-form gold nanostructures and tetrahedral DNA probes. Furthermore, aptamer biosensor technologies, which mainly consist of optical and electrochemical aptasensors, can also enable the rapid identification of microorganisms(Yi et al., 2020). Although they perform well in buffer systems, their practical application is limited by the non-specific interference and aptamer structural changes in complex sample conditions. In conclusion, integrating nanozymes with electrochemical biosensors presents a novel technique for biosensing, with the capacity to markedly enhance the sensitivity, selectivity, and stability. However, non-specific adsorption, signal crosstalk in multi-analyte detection, and complicated instrumentation still limit their application in on-site diagnosis.

### 3.3 Fluorescent and dual-modal sensing

Fluorescent and dual-modal sensors combine nanozymes with fluorescent probes to secure high sensitivity and specificity in the detection process. For example, a dual-modal sensor for nitrite using Mn-CDs achieved an LOD of 3.5 nM using fluorescence and 73 nM using colorimetry (Wang et al., 2022). Another study, a multivalent aptamer-driven “4 + 1” nanozyme biosensing platform, which combined exponential amplification reaction, hybridization chain reaction, multivalent aptamers, and dual nanozymes, was developed for fluorescence-colorimetric dual-modal detection of *Salmonella typhimurium* (Kang et al., 2026). This method achieved LOD of 7.7 CFU/mL for fluorescence mode and 8.7 CFU/mL for colorimetric mode. Furthermore, a target-inhibited colorimetric/fluorescence/SERS trimodal biosensor, which relied on target-inhibited electrostatic self-assembly between magnetic covalent organic frameworks (MCOF) and aptamer-functionalized Ag nanozymes, was developed for detection of *Vibrio parahaemolyticus* (Xiao et al., 2026). After the optimization process, the sensor achieved an LOD of 1.50 CFU/mL for colorimetry, 1.02 CFU/mL for fluorescence, and 0.97 CFU/mL for SERS. In conclusion, the integration of fluorescent and dual-modal sensing technologies represents a significant advancement in analytical methodologies. Nevertheless, this sensing strategy often requires complicated signal readout equipment, and multiple signal channels may suffer from mutual interference (see Table 1).

**Table 1:** Methods for detecting microorganisms by nanozyme-based biosensors.

Nanozyme material	Catalytic class	Target pathogen	Matrix tested	Assay time	LOD	Instrument requirement	References
CTAB-AuNPs	Peroxidase-like nanozyme activity	*E. coli*	Pasteurized milk samples	80 min	15 CFU/mL	UV–Vis spectrophotometer	Wang et al. (2023)
Au@Fe/ZIF	Dual-nanozyme cascade activity	*B. melitensis* and *B. cereus*	Water from dehumidifier, refrigerator, humidifier, water dispenser; soil soaking extracts; swab eluent from air-conditioner surfaces.	35 min	Cascade colorimetric nanozyme mode: 10 CFU/mL; SERS mode: 10 CFU/mL	UV–Vis spectrophotometer, confocal raman spectrometer, and microfluidic chip platform	Su et al. (2026)
Fe-PDs	Peroxidase-like nanozyme activity	*S. typhimurium*	Milk	145 min	7.7 CFU/mL for fluorescence and 8.7 CFU/mL for colorimetry	Fluorescence spectrophotometer and UV–Vis spectrophotometer	Kang et al. (2026)
MCOF-Apt@Ag	Peroxidase-like nanozyme activity	*V. parahaemolyticus*	Shrimp, salmon	75 min	1.50 CFU/mL for colorimetry, 1.02 CFU/mL for fluorescence, 0.97 CFU/mL for SERS	UV–Vis spectrophotometer, Fluorescence spectrophotometer, and high-resolution confocal Raman spectrometer	Xiao et al. (2026)
NH_2_-MnO_2_-NRs	Oxidase-like nanozyme activity	SARS-CoV-2	Pseudovirus samples	100 min	10 copies/μL	UV–Vis spectrophotometer or microplate reader	Wu et al. (2022)
CS-PtNCs	Peroxidase-like nanozyme activity	*S. aureus*	Cerebrospinal fluid	60 min	20 CFU/mL	UV–Vis spectrophotometer or microplate reader	Yi et al. (2026)
AIE MOFzyme	Peroxidase-like nanozyme activity	*S. enterica*	Milk, egg, meat	110 min	1 CFU/mL for fluorescence and 15.9 CFU/mL for colorimetry	Fluorescence microplate reader and UV–Vis spectrophotometer	Qiu et al. (2025)
PCN-224-Mn	Oxidase-like nanozyme activity	*K. pneumoniae* and *A. baumannii*	Human serum, urine, sputum	40 min	5 CFU/mL for *K. pneumoniae* and 7 CFU/mL for *A.r baumannii*	UV–Vis spectrophotometer	Li et al. (2026)

### 3.4 Nanozyme-CRISPR synergistic detection

By merging the sequence-specific recognition of CRISPR-Cas systems with the signal-amplification capability of nanozymes, their integration has become a transformative strategy for biosensing. The hybrid platforms can achieve unprecedented levels of sensitivity and specificity. Chen et al. developed an Au-Fe₃O₄ nanozyme-CRISPR-Cas12a platform to identify antibiotic resistance genes such as those for chloramphenicol, kanamycin, and ampicillin, thereby achieving an LOD < 0.1 CFU/μL (Chen et al., 2023). In another research, a CRISPR-Cas12a-MXene nanozyme platform was utilized for detecting trace DNA, reaching an LOD of 132 copies/μL (Liu et al., 2026). Nanozyme-CRISPR systems can be used for more than detecting nucleic acids. For instance, a MnO_2_ nanozyme-mediated CRISPR-Cas12a system has been developed for the detection of SARS-CoV-2 (Wu et al., 2022). This system utilizes MnO_2_ nanorods linked to magnetic beads, which induce a color change upon activation by the target virus, enabling visual detection. The high selectivity, anti-interference capability, ultra-low LOD values, and ease of operation make this platform suitable for POCT, highlighting the potential of nanozyme-CRISPR systems in pandemic response. With ongoing research, the creation of advanced biosensors utilizing the distinct characteristics of nanozymes and CRISPR-Cas systems is expected to result in more effective and user-friendly diagnostic tools. However, the strict reaction condition, sophisticated operation procedures, and complex reagent system are key persistent limitations.

## 4 Typical applications of nanozyme-based biosensors in pathogenic microorganism detection

### 4.1 Clinical pathogens diagnosis

Nanozyme-based biosensors have revolutionized clinical pathogen diagnosis by enabling rapid, sensitive detection of infectious agents. For instance, Yi et al. developed a cationic platinum nanocluster-based loop-mediated isothermal amplification platform to detect MRSA (Yi et al., 2026). This assay supports detection through both visual and colorimetric means, achieving a low LOD of 20 CFU/mL in clinical cerebrospinal fluid (CSF) samples. Owing to remarkable resilience and high transmissibility, viruses represent major targets in biosafety management. The magnetic nanozyme-linked immunosorbent assay (MagLISA), a nanozyme-based colorimetric immunosorbent assay, was developed to enable one-step identification, separation and visualization of influenza viruses. The system achieved an ultra-low limit of detection of 10^−14^ g·mL^−1^ when paired with commercial microreaders (Oh et al., 2018). A triple-modal sensing platform, which integrated the high-performance AFRNBs@PtNPs nanozyme and the XGBoost-SHAP-based AI strategy, was developed for the detection of *S. typhimurium* with an LOD of 204 CFU/mL (Li et al., 2025). In conclusion, nanozyme-based biosensors represent a significant advancement in the field of clinical pathogen diagnosis. Their unique properties and integration into various biosensing platforms have enabled the fast, precise, and specific detection of pathogenic microorganisms, addressing critical needs in clinical diagnostics.

### 4.2 Foodborne pathogenic bacteria detection

The detection of foodborne pathogenic bacteria is essential for controlling and preventing food-safety risks. However, factors including complex food matrices, low target concentrations and the co-existence of multiple pathogenic bacteria, continue to present significant technical challenges for the effective detection. Foodborne pathogen detection is a key application of nanozyme-based biosensors, addressing the need for rapid, on-site monitoring. Qiu et al. developed a CRISPR-responsive DNA hydrogel biosensor for *S. enterica* detection with an LOD of 1 CFU/mL for fluorescence and 15.9 CFU/mL for colorimetry (Qiu et al., 2025). This study reported a dual functional AIE MOFzyme nanozyme, which has both aggregation-induced emission fluorescence and peroxidase mimicking activities, was encapsulated in a DNA hydrogel derived from rolling circle amplification. The study of Ag^+^-gated peroxidase activity of AuNPs for the sensitive detection of *E. coli* represents another significant advancement in the field of biosensing, particularly for foodborne pathogens (Wang et al., 2023). The fundamental concept of this technique is based on altering the peroxidase-like activity of AuNPs through the interaction with Ag^+^, which disrupts the cetyltrimethylammonium bromide membrane on the surface of the nanoparticles. The study demonstrates a linear relationship between the absorbance intensity and the concentration of *E. coli*, ranging from 1.0 × 10^2^ to 1.0 × 10^9^ CFU/mL, highlighting its potential for practical applications in pathogen detection. Therefore, the ability of nanozyme-based biosensors to provide fast, sensitive, and accurate detection of pathogens makes them invaluable tools in the food industry.

### 4.3 Environmental microbial monitoring

Antimicrobial resistance is a major threat to global public health. Antibiotic-resistant bacteria and their resistance genes are persistently spreading in environment, which further leads to refractory bacterial infections in humans. Therefore, environmental monitoring plays an irreplaceable role in delivering early warnings for potential AMR transmission from the environment to humans. Nanozyme-based biosensors have emerged as a transformative technology for monitoring environmental microbes in air, water, and soil. Li et al. designed an aptamer-based “gate-lock-key” colorimetric biosensing platform, which relies on UiO-66-NH₂ MOFs and PCN-224-Mn nanozyme, to detect multidrug-resistant bacteria (Li et al., 2026). When the target bacteria are present, they induce the dissociation of the aptamer-ssDNA duplex on the MOF surface. And then, encapsulated TMB was released and oxidized by the PCN-224-Mn nanozyme to generate a visible color signal. This method achieves LOD as low as 5 CFU/mL for *K. pneumoniae* and 7 CFU/mL for *Acinetobacter baumannii*. This aptamer-gated sensing approach provides a practical method for rapid detection of multidrug-resistant bacteria. Moreover, an innovative platform integrates nanozyme cascade colorimetric reactions, surface-enhanced Raman spectroscopy, and photothermal sterilization, offering a comprehensive approach to detecting and inactivating bacterial contaminants in complex environments. The system achieved an LOD as low as 10 CFU/mL for both *Brucella melitensis* and *Bacillus cereus*, which underscores its potential for applications in environmental microbial monitoring (Su et al., 2026). Meanwhile, nanozymes have also been applied for the detection of environmental drug resistance genes. Through combining apatamer, CRISPR-Cas12a and magnetic separation, Zhang et al. developed a Prussian-blue@UiO-66@MnO₂ based luminescent nanozyme biosensor to detect ampicillin and β-lactam antibiotic resistance genes (Zhang T. et al., 2025). Overall, the integration of nanozyme-based biosensors in environmental microbial monitoring offers a promising avenue for addressing critical challenges in public health and environmental protection.

## 5 Conclusions and future perspectives

In summary, nanozymes have shown remarkable potential due to their high stability, tunable catalytic activity, and cost-effectiveness compared to natural enzymes. These characteristics render them excellent choices for biosensor applications in diverse areas such as healthcare, food safety, and environmental monitoring. However, nanozyme-based pathogenic microbial detection still faces some limitations, including insufficient catalytic selectivity, severe matrix interference from practical samples, and restricted capacity for simultaneous multi-target detection at current stage. These limitations result in limited applications of nanozyme-based biosensors. In the future, AI aided design and modification of nanozymes are important research directions. On the one hand, the clinical and industrial translation of nanozyme-based biosensors driven by AI holds significant promise for revolutionizing diagnostic and therapeutic monitoring. First, AI technologies, particularly machine learning (ML), facilitate the rational prediction of catalytic activities and the optimization of nanozyme structures, thereby enhancing their performance in biosensor applications. Furthermore, AI-driven methodologies are crucial for addressing the challenges associated with the clinical translation of nanozyme-based biosensors, such as scalability, regulatory compliance, and the interpretability of computational models.
